# Complete chloroplast genome sequence of *Petrocodon jingxiensis* (Gesneriaceae)

**DOI:** 10.1080/23802359.2019.1624208

**Published:** 2019-07-29

**Authors:** Zi-Bing Xin, Long-Fei Fu, Zhi-Xi Fu, Shu Li, Yi-Gang Wei, Fang Wen

**Affiliations:** a Guangxi Key Laboratory of Plant Conservation and Restoration Ecology in Karst Terrain, Guangxi Institute of Botany, Guangxi Zhuang Autonomous Region and, Chinese Academy of Sciences, Guilin, China;; b Gesneriad Conservation Center of China, Guilin Botanical Garden, Chinese Academy of Sciences, Guilin, China;; c Laboratory of Systematic Evolution and Biogeography of Woody Plants, College of Nature Conservation, Beijing Forestry University, Beijing, China;; d College of Life Sciences, Sichuan Normal University, Chengdu, China

**Keywords:** Petrocodon, plastid genome, phylogeny, Gesneriaceae

## Abstract

The complete chloroplast genome of *Petrocodon jingxiensis* (Yan Liu, H.S. Gao, and W.B. Xu) A. Weber and Mich. Möller was determined in this study. The cp genome was 153,056 bp in length including two inverted repeats (IRs) of 25,405 bp, which were separated by LSC and SSC of 84,154 bp and 18,092 bp, respectively. The GC content was 41.9%. The genome encoded 112 functional genes, including 79 protein-coding genes, 29 tRNA genes, and four rRNA genes. This plastid genome is the first report for the *Petrocodon* in Gesneriaceae which will be useful data for developing markers for further studies on resolving the relationship within the genus.

*Petrocodon* Hance (Gesneriaceae), is a small genus growing in mountainous karst habitats of S China, N Vietnam and NE Thailand (Hance 1883; Wang et al. 1998; Li and Wang 2004; Wei et al. 2010). Based on the latest molecular phylogenetic analyses, *Petrocodon* has been expanded to now include more than 30 species (Wang et al. 2011; Weber et al. 2011; Möller et al. 2016; IPNI 2019), and include all species previously referred to the genera *Calcareoboea* C. Y. Wu ex H. W. Li, *Paralagarosolen* Y. G. Wei, *Dolicholoma* D. Fang and W. T. Wang, *Tengia* Chun, and *Lagarosolen* W. T. Wang, and four species from *Didymocarpus* Wall., one species from *Wentsaiboea* D. Fang and D. H. Qin and one species from *Primulina* Hance (Weber et al. 2011). Despite Möller et al. (2016) reconstructed the phylogenetic relationship of Gesneriaceae, the relationships within *Petrocodon* still remain poorly resolved.

The DNA sequence of the chloroplast genome can be used as a super barcode or a resource for research in phylogeograhy, genetic diversity and evolution. For *Petrocodon*, however, no complete chloroplast sequence has been published to date.

In the present study, mature and healthy leaves of *Petrocodon jingxiensis* were collected from natural population in Jingxi County, Baise City, Guangxi, China (N 106°21′24″, E 23°1′32″) and immediately dried by silica gel for DNA extraction. Voucher specimen (WF046) of this collection was deposited at IBK. The total genomic DNA was extracted using the CTAB method (Doyle and Doyle 1987) with minor modification and we sequenced the complete chloroplast genome of *Petrocodon jingxiensis* with Illumina Hiseq 4000 sequencing platform (Novogene, http://www.novogene.com, Beijing, China). We used Map to Reference function in Geneious R11 (Kearse et al. 2012) to exclude nuclear and mitochondrial reads using published plastid genome of *Primulina huajieensis* (MF472012) as reference. The cp genome was manually adjusted to remove ambiguous sites. The annotation process was performed following Liu et al. (2018) using *Primulina huajieensis* (MF472012) as the reference. The complete chloroplast genome of *Petrocodon jingxiensis* was 153,056 bp in length (MK887172), the GC content was 41.9%. LSC and SSC contained 84,154 bp and 18,092 bp respectively, while IR was 25,405 bp in length. The plastid genome encoded 112 functional genes, including 79 protein-coding genes, 29 tRNA genes, and four rRNA genes.

The maximum likelihood phylogenetic analysis of 10 chloroplast genomes showed that *Petrocodon jingxiensis* was most closely related to the members of *Primulina* (Figure 1). The newly characterized cp genome of *Petrocodon jingxiensis* will provide essential data for further study on the phylogeny and evolution of the genus *Petrocodon* and of the family Gesneriaceae.

**Figure 1.**
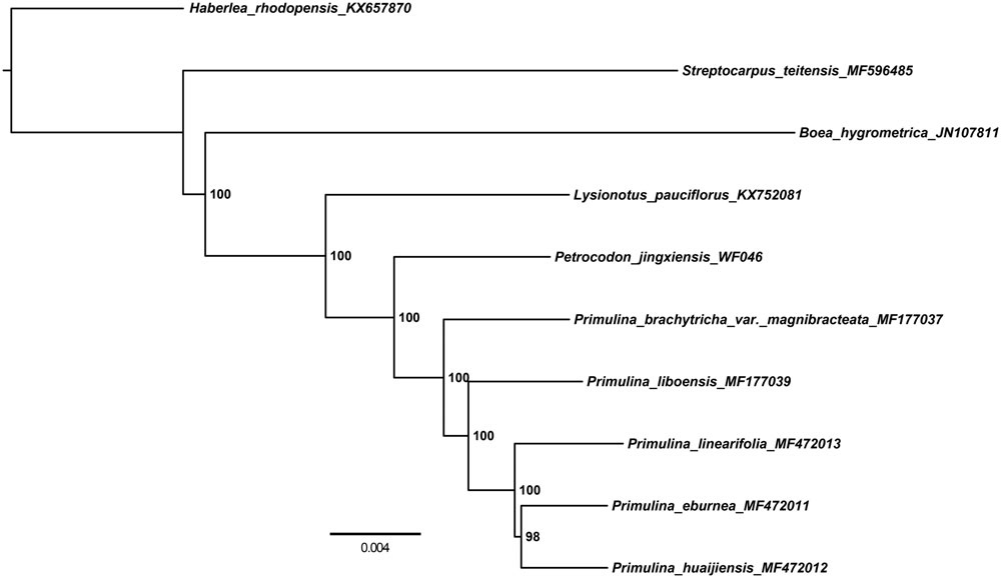
Phylogenetic tree reconstructed by maximum-likelihood (ML) analysis based on chloroplast genome sequences from 10 species of Gesneriaceae, numbers next to notes are assessed by ML bootstrap.

## References

[CIT0001] Doyle JJ, Doyle JL 1987 A rapid DNA isolation procedure for small quantities of fresh leaf material. Phytochem Bull. 19:11–15.

[CIT0002] Hance HF 1883 New Chinese Cyrtandreae. J. Bot. 21:169.

[CIT0003] IPNI 2019 The International Plant Names Index. http://www.ipni.org [accessed 2019 Apr 23].

[CIT0004] Kearse M, Moir R, Wilson A, Stones-Havas S, Cheung M, Sturrock S, Buxton S, Cooper A, Markowitz S, Duran C, et al. 2012 Geneious Basic: an integrated and extendable desktop software platform for the organization and analysis of sequence data. Bioinformatics. 28:1647–1649. 22543367 10.1093/bioinformatics/bts199 PMC3371832

[CIT0005] Li ZY, Wang YZ 2004 Plants of Gesneriaceae in China. Zhengzhou: Henan Science and Technology Publishing House; p. 170–260.

[CIT0006] Liu H, He J, Ding C, Lyu R, Pei L, Cheng J, Xie L 2018 Comparative analysis of complete chloroplast genomes of *Anemoclema*, *Anemone*, *Pulsatilla*, and *Hepatica* revealing structural variations among genera in tribe Anemoneae (Ranunculaceae). Front Plant Sci. 9:1097. 30100915 10.3389/fpls.2018.01097 PMC6073577

[CIT0007] Möller M, Wei YG, Wen F, Clark JL, Weber A 2016 You win some you lose some: updated generic delineations and classification of Gesneriaceae – implications for the family in China. Guihaia. 36:44–60.

[CIT0008] Wang YZ, Mao RB, Liu Y, Li JM, Dong Y, Li ZY, Smith JF 2011 Phylogenetic reconstruction of *Chirita* and allies (Gesneriaceae) with taxonomic treatments. J Syst Evol. 49:50–64.

[CIT0009] Wang WT, Pan KY, ZY L. 1998 Gesneriaceae In: Wu ZY and Raven PH, editors. Flora of China 18. Beijing: Science Press; China and Botanical Garden Press, St. Louis, Missouri; p. 244–401.

[CIT0010] Weber A, Wei YG, Puglisi C, Wen F, Mayer V, Möller M 2011 A new definition of the genus *Petrocodon* (Gesneriaceae). Phytotaxa. 23:49–67.

[CIT0011] Wei YG, Wen F, Möller M, Monro A, Zhang Q, Gao Q, Mou HF, Zhong SH, Cui C 2010 Gesneriaceae of South China. Zhengzhou: Henan Science and Technology Publishing House; p. 274–527.

